# Deletion in the *EVC2* Gene Causes Chondrodysplastic Dwarfism in Tyrolean Grey Cattle

**DOI:** 10.1371/journal.pone.0094861

**Published:** 2014-04-14

**Authors:** Leonardo Murgiano, Vidhya Jagannathan, Cinzia Benazzi, Marilena Bolcato, Barbara Brunetti, Luisa Vera Muscatello, Keren Dittmer, Christian Piffer, Arcangelo Gentile, Cord Drögemüller

**Affiliations:** 1 Institute of Genetics, Vetsuisse Faculty, University of Bern, Bern, Switzerland; 2 Department of Veterinary Medical Sciences, University of Bologna, Ozzano dell'Emilia, Italy; 3 Institute of Veterinary, Animal and Biomedical Sciences, Massey University, Palmerston North, New Zealand; 4 Servizio Veterinario dell'Azienda Sanitaria dell'Alto Adige, Bozen, Italy; University of Sydney, Australia

## Abstract

During the summer of 2013 seven Italian Tyrolean Grey calves were born with abnormally short limbs. Detailed clinical and pathological examination revealed similarities to chondrodysplastic dwarfism. Pedigree analysis showed a common founder, assuming autosomal monogenic recessive transmission of the defective allele. A positional cloning approach combining genome wide association and homozygosity mapping identified a single 1.6 Mb genomic region on BTA 6 that was associated with the disease. Whole genome re-sequencing of an affected calf revealed a single candidate causal mutation in the Ellis van Creveld syndrome 2 (*EVC2*) gene. This gene is known to be associated with chondrodysplastic dwarfism in Japanese Brown cattle, and dwarfism, abnormal nails and teeth, and dysostosis in humans with Ellis-van Creveld syndrome. Sanger sequencing confirmed the presence of a 2 bp deletion in exon 19 (c.2993_2994ACdel) that led to a premature stop codon in the coding sequence of bovine *EVC2*, and was concordant with the recessive pattern of inheritance in affected and carrier animals. This loss of function mutation confirms the important role of *EVC2* in bone development. Genetic testing can now be used to eliminate this form of chondrodysplastic dwarfism from Tyrolean Grey cattle.

## Introduction

Dwarfism in calves is a congenital anomaly whereby affected animals can be differentiated from normal animals on the basis of relative length of the limbs (OMIA 000299-9913). Proportional dwarfism in cattle, so called miniature calves, has been reported several times (OMIA 000308-9913, OMIA 001485-9913, OMIA 001686-9913, OMIA 001473-9913). In a family of Holstein cattle it has been associated with down-regulation of insulin-like growth factor 1 [1], while in Brahman, a mutation within the *GH1* gene is associated with the miniature condition [3]. A mutation in the *RNF11* gene leads to dwarfism in Belgian Blue cattle in association with compromised resistance to pathogens [2]. Congenital disproportionate dwarfism, designated as chondrodysplasia, achondroplasia or chondrodystrophy, is described in a number of different cattle breeds with varying modes of inheritance (OMIA 000004-9913, OMIA 000187-9913, OMIA 000311-9913, OMIA 001271-9913, OMIA 000189-9913). Congenital chondrodystrophy of unknown origin has been reported in beef cattle worldwide indicating possible non-genetic causes, such as abnormal nutrition, can be involved in some cases [4], [5]. The different types of bovine chondrodysplastic dwarfism show varying levels of severity, ranging from lethal and semi-lethal to viable forms. Chondrodysplasia is primarily caused by defects in genes that regulate normal chondrogenesis and cartilage development, resulting in abnormal shape and structure of the skeleton. The molecular basis of a number of bovine chondrodysplasias has been determined. Bulldog dwarfism in Dexter cattle, one of the earliest described Mendelian disorders of animals, is associated with a 4 bp insertion in the coding region of the aggrecan (*ACAN*) gene. This disease has incompletely dominant inheritance, leading to a mild form of dwarfism in heterozygotes, while homozygous animals display extreme disproportionate dwarfism and usually die during gestation [6]. An outbreak, with striking similarities to bulldog dwarfism, has also been reported in the offspring of a single internationally used Holstein sire [7]. This form of chondrodysplastic dwarfism is probably caused by a still unpublished dominant *de novo* mutation (http://www.labogena.fr/). A mutation in the kinase *PRKG2* is associated with autosomal recessive long-nosed dwarfism in Angus cattle [8]. Chondrodysplastic dwarfism also occurs in small ruminants; causative mutations in two genes, *FGFR3* and *SLC13A1*, have been identified as causing recessively inherited forms of chondrodysplasia in Suffolk and Texel sheep, respectively [9], [10].

Chondrodysplastic dwarfism in Japanese Brown cattle was determined to be due to a mutation in the *Limbin* gene [11]. This gene has since been renamed Ellis van Creveld syndrome 2 (*EVC2*) gene, as it has been suggested that it arose by duplication of the immediately adjacent Ellis van Creveld syndrome (*EVC*) gene [12], [13]. Both genes are associated with forms of Ellis-van Creveld syndrome in humans (OMIM 225500). Ellis-van Creveld syndrome is an autosomal recessive disorder in which affected individuals have chondrodysplasia, polydactyly, nail dysplasia, orofacial abnormalities and, in a proportion of patients, cardiovascular malformations. The phenotype associated with the mutations in either *EVC* or *EVC2* is indistinguishable [12]–[14]. Weyers acrofacial dysostosis (OMIM 193530), an autosomal dominant disorder with a similar but milder phenotype, is also associated with the *EVC* genes [15].

Tyrolean Grey cattle, or Grauvieh, are a dual-purpose (bred for milk and meat) alpine cattle breed. The population is small with only a few thousand registered cows, predominantly in Austria (Tyrol) and Italy (South Tyrol), and lower numbers in Switzerland. In the last decade a Tyrolean Grey cattle breeder has experienced more than 50 cases of a recessively inherited degenerative axonopathy. A causative mutation in the *MFN2* gene was identified three years ago, and gene testing to eliminate the disease from the population is ongoing [16].

During the summer of 2013 in South Tyrol, seven inbred calves were born with abnormally short limbs. The goal of the present study was to quickly identify the causative mutation for the condition using massive genotyping and next generation sequencing, in order to develop a genetic test so that further risk mating could be prevented.

## Results and Discussion

### An outbreak of a recessive defect in Tyrolean Grey cattle

**Figure 1:**
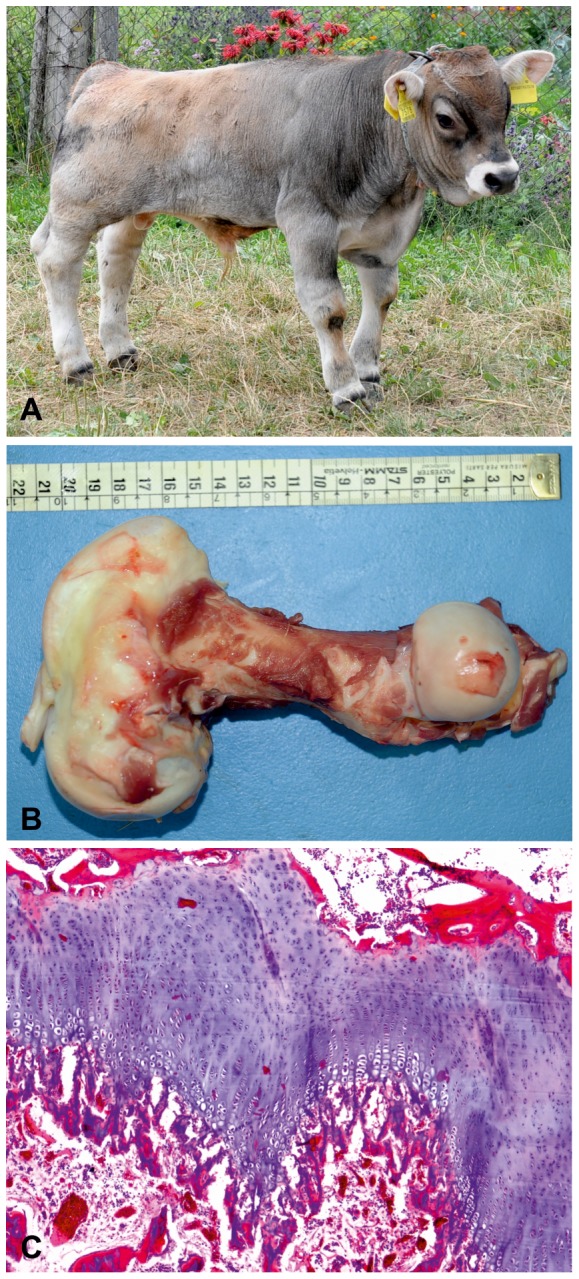
Dwarf Tyrolean Grey calf phenotype. (**A**) Seven month old affected Tyrolean Grey calf (1) and five month old normal control (2). Note the shorter legs and twisted posture. The head proportions and axial skeleton appear to be normal. At the moment the photo shoot both animals show an identical femur width of 3 cm and the distance between caput ossis femoris and trochlea ossis femoris was 20.9 cm for the affected and 26.8 cm in the control. (**B**) The femur is shortened and moderately twisted, with enlargement of, particularly the distal metaphysis and epiphysis. The scale is shown for reference. (**C+D**) X-ray of tibia from a normal and an affected animal. The ruler corresponds to 10 cm. (**E+F**) Histology of the femur of normally developed control of same age and an affected animal showing irregularity of the growth plate and increased thickness of the reserve zone (H&E 4x). Both the proliferative and hypertrophic zones are shortened and disorganized. Trabeculae in the primary spongiosa are also truncated.

**Figure 2:**
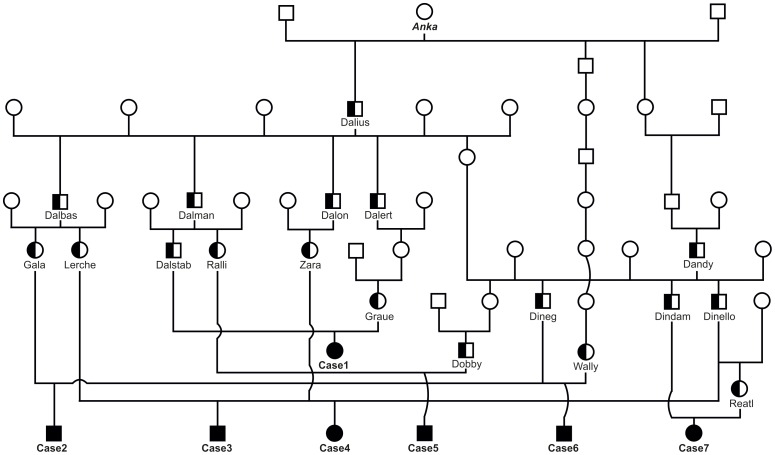
Family tree of seven Tyrolean Grey cattle with chondrodysplastic dwarfism. Males are represented by squares, females by circles. Affected animals are shown with fully black symbols and genotyped carriers with a half-filled symbol.Animals without names, not available for genotyping, are shown with empty symbols. The mutation probably occurred in the cow *Anka* born in 1982 (no DNA available) and was spread into the population by her son *Dalius* (an acknowledged carrier) and at least two other descendants. All parents of affected calves are related to this cow.

During July to October 2013, a total of 7 Tyrolean Grey calves, 4 males and 3 females, aged 1 week to 2.5 months, were presented to the authors with disproportionate dwarfism due to shortening of all limbs (**Figure 1**). The parents of all cases were of normal size. Analysis of the pedigree data revealed that three paternal half-sibs, used as artificial insemination sires (Dineg, Dindam, and Dinello), had five affected offspring among their progeny. Furthermore, all 7 dams and the 2 sires of the remaining two cases belonged to the same breeding line. All parents could be traced back (over 2 to 7 generations) to a single common female ancestor (Anka) born in 1982 (**Figure 2**). Therefore, we concluded that a recessively inherited mutation was most likely. These findings provide a second example of how the current breeding practices in numerically small cattle breeds like Tyrolean Grey may lead to the outbreak of a genetic disease. As reported for the degenerative axonopathy, a single intensively used animal has transmitted the deleterious allele to all cases within a few generations [16].

### A new form of bovine chondrodysplastic dwarfism

Four affected animals were admitted to the Department of Veterinary Medical Sciences at the University of Bologna for in-depth clinical study. Two calves are still alive, while the other two were euthanized and underwent a post-mortem examination. All patients were bright and alert but had difficulty rising and/or maintaining a quadrupedal stance. The limbs were disproportionately short and bulky, variably rotated, and arched in a dumbbell-like position. The abnormal axial position of the limbs, together with moderate to pronounced joint laxity, prevented efficient support of weight. Joint laxity was also likely responsible for the wobbling gait shown by affected calves when walking. One calf had bilateral hyperextension of all hindlimb joints due to spastic contraction of limb muscles. Another calf had a caudally declining dorsal profile resembling hyena disease [17]. The splanchnocranium, mandible and palate were normal, as was the heart, and there was no polydactyly. Radiographs of one hospitalized calf showed very short, twisted long bones, however the bones were not osteopenic and there were no fractures. The femur and humerus were most severely shortened; conducting measurements on the necropsy sample the femur was 21 cm long, with a diaphyseal length of 8 cm, and the humerus 15 cm long with a diaphyseal length of 5.5 cm (**Figure 1**). In the second calf the femur was 18.5 cm long, with a diaphyseal length of 5 cm, and the humerus was 12 cm in length, with a diaphyseal length of 4 cm.

Histopathological examination of both affected calves revealed that the growth plate of long bones and vertebrae, which were grossly normal, was irregular and had closed prematurely (**Figure 1**). The reserve zone was extremely variable in thickness. Chondrocytes in the proliferative and hypertrophic zones were disorganized, had multifocal loss of the normal columnar arrangement, and were haphazardly arranged individually or in nests (**Figure 1**). Some chondrocytes contained cytoplasmic vacuoles. The metaphysis was reduced in length, and trabeculae in the primary spongiosa were shortened (**Figure 1**).

The genital tracts appeared fully mature, despite the 2 examined female calves being only 2 and 2.5 months old. There were numerous follicle-like structures on the ovaries, and the uterus had multiple endometrial polyps (**Figure S2**). Within the ovaries, primordial, primary, secondary and vesicular follicles were present, as were rare corpora lutea. The uteri contained multiple polypoid structures, characterized by a core of mature collagen lined by endometrial epithelium. Clinically, male genitalia size and morphology was not different to that expected for animals of a similar age and breed.

### Mapping to a region on BTA 6 containing functional candidate genes

**Figure 3:**
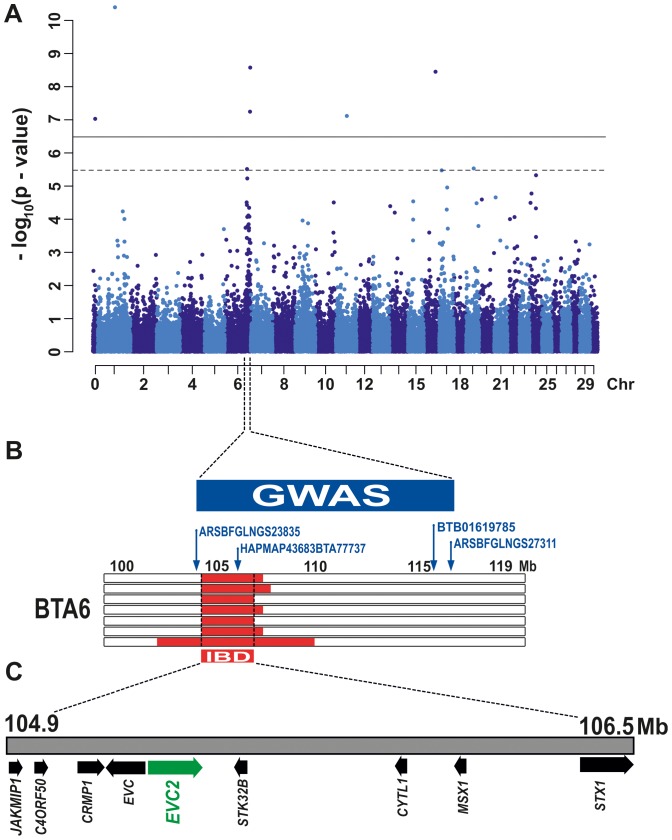
Mapping to BTA 6. (**A**) Results of the genome-wide association study (GWAS): Manhattan-plot showing the negative log of the raw P values calculated with the mixed-model genotypic association test. Genome-wide significance thresholds are indicated (P≤0.01, solid horizontal line; P≤0.1 dotted horizontal line). (**B**) Detailed localization of the most significantly associated BTA 6 SNPs in combination with the identical by descent (IBD) segments of the seven genotyped affected animals. The codes of SNPs over the threshold lines are reported along with their positions on the map. (**C**) Gene content of the fine-mapped critical interval.

We genotyped 777,962 SNPs on 7 affected calves. As controls, 44 Tyrolean Grey cattle were genotyped for 54,001 evenly spaced SNPs which had been generated in the course of a previous study [16]. A total of 43,646 SNPs was genotyped in both cohorts. After removing 12,601 non-informative markers, 31,045 SNPs were used for genome-wide association mapping (GWAS). The genomic inflation factor in this analysis was 2.29; a high value caused by the use of highly stratified and sometimes closely related animals. Therefore we performed a mixed-model analysis to control for the population stratification, and this showed 20 significantly associated markers (raw P values <10^−5^; **Table S1**). A total of 15 of these SNPs were located individually, at different genomic regions, on 12 different chromosomes. This is likely due to independent genotyping of case and control samples on different arrays at different times. Nonetheless, we detected a total of 4 highly significantly associated SNPs, in a single contiguous genomic region (103–116 Mb), on bovine chromosome 6 (BTA 6; **Figure S1**). The association of 2 out of 4 SNPs exceeded the threshold for genome-wide significance after Bonferroni correction (P≤0.01), and the other 2 a lesser stringed adjustment of P≤0.1 Therefore we considered a potential candidate region for chondrodysplastic dwarfism (**Figure 3**). This analysis was followed by a homozygosity mapping approach, to fine-map the region containing the responsible mutation. Based on the pedigree records we hypothesized that affected calves were most likely inbred, and related to a single founder animal (**Figure 2**). Under this scenario the affected individuals were expected to be identical by descent (IBD) for the causative mutation and flanking chromosomal segments. We analyzed the cases looking for extended regions of homozygosity with simultaneous allele sharing and found a single common region in the genome that fulfilled our search criteria. On BTA 6 all the seven cases were homozygous, and shared identical alleles over 627 SNP markers, corresponding to a 1.6 Mb interval from BTA 6:104,916,545 to 106,486,864 (**Figure 3**). Taken together, these mapping efforts indicated that the responsible mutation causing chondrodysplastic dwarfism in Tyrolean Grey cattle mapped to a region on BTA 6. The mapped interval contains nine annotated genes (Figure 3) and a careful inspection of these genes and database searches of their presumed function revealed the three genes encoding Ellis van Creveld syndrome (*EVC*), Ellis van Creveld syndrome 2 (*EVC2*) and Msh homeobox 1 (*MSX1*) were possible candidate genes. Heritable human Ellis-van Creveld syndrome and Weyers acrofacial dysostosis, belong to a group of diseases known as ciliopathies, and are caused by mutations in the *EVC* and *EVC2* genes. These mutations result in aberrant responses to the hedgehog ligands required for normal endochondral growth [14], [18]. Msx homeodomain transcription factors play important roles in the control of limb development [19].

### A deletion in the *EVC2* gene causes chondrodysplastic dwarfism

**Figure 4:**
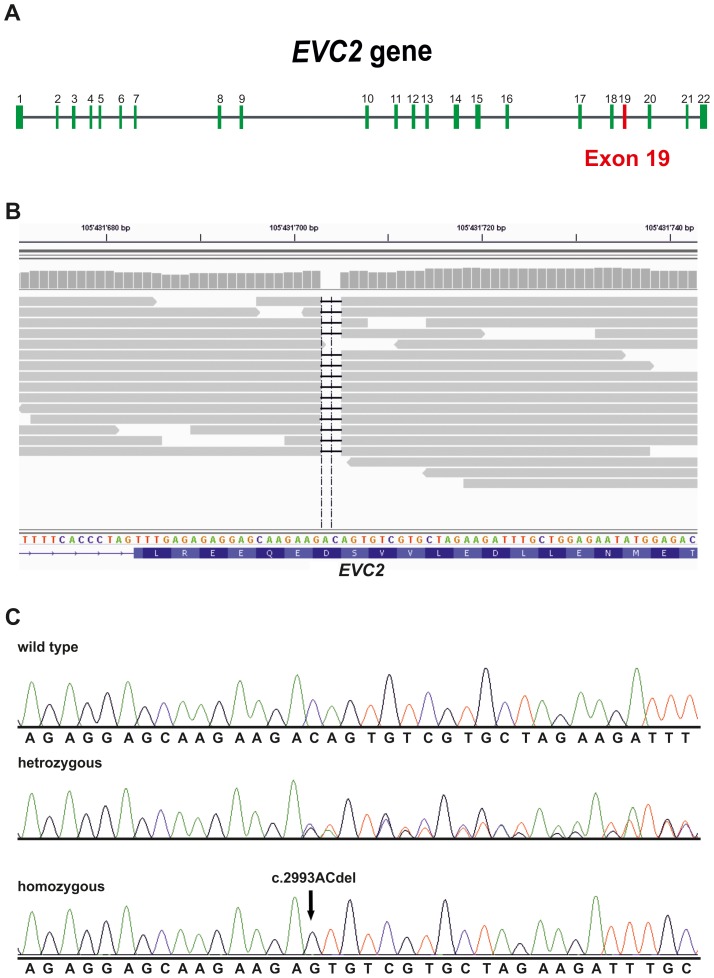
Mutation detection by whole genome re-sequencing and subsequent Sanger sequencing. (**A**) *EVC2* gene (**B**) Screenshot of the next generation sequence reads mapped against the reference sequence. Note the straight black line indicating the two base pair deletion, and the translated reference sequence below. (**C**) Sanger sequencing confirmed the presence of the genomic deletion. Electropherograms of a control Tyrolean Grey cattle (wild type), a parent of one of the affected calves (heterozygous), and one affected animal (homozygous).

We opted to sequence the whole genome of one affected calf (case 1, **Figure 2**) and then focus on the critical zone in chromosome 6 to detect all the variants in the annotated genes and loci. We collected 178 million 2×100 bp paired-end reads from a shotgun fragment library corresponding to roughly 13× coverage of the genome. SNPs and short indel variants were called with respect to the reference genome and detected 119,998 high quality variants across the whole genome, of which 32,996 were coding variants. Due to the recessive inheritance and the fatal effect of the mutation, we hypothesized that a loss of function mutation affecting the coding sequence of one of the functional candidate genes would be responsible for chondrodysplastic dwarfism. Within the mapped 1.6 Mb interval, we detected 151 coding variants located within the coding sequences or within the splice sites of the annotated genes in the targeted region, of which 35 were predicted to affect the amino acid sequence (**Table S2**). Therefore, we subsequently concentrated on these 35 variants. Comparison between the affected calf and 28 cow genomes of various breeds that had been sequenced in our laboratory in the course of other studies (**Table S3**) revealed that 34 of these variants occurred in the control genomes and could thus be excluded as causative variants. The remaining variant was a 2 bp deletion located in exon 19 of the bovine *EVC2* gene (c.2993ACdel, **Figure 4**). This variant was validated by Sanger sequencing, which subsequently confirmed that the affected calf was homozygous, while the parents were heterozygous when compared to the reference sequence (**Figure 4**). There was100% concordance between the presence of this deletion and the dwarfism phenotype (**Table 1**). All seven affected calves were homozygous mutant and all 12 parents were heterozygous. None of 913 healthy Tyrolean Grey cattle genotyped by Sanger sequencing had the homozygous mutant genotype, but 28 of them were determined to be carriers. Thus the allele frequency of the deleterious insertion within this sample of Tyrolean Grey cattle was 1.5%. Interestingly, all 40 heterozygous carriers belonged exclusively to the Italian population of Tyrolean Grey cattle, and the current carrier frequency in this population is close to 15%. In addition, available ancestors of the seven cases were shown to be carriers thus confirming the initial suspicion that a recent mutation event in the common female ancestor (Anka) was responsible for the outbreak (**Figure 2**).

**Table 1:** Association of the 2*EVC2* gene with the chondrodysplastic dwarfism phenotype.

	wt/wt	wt/del	del/del
Affected calves	-	-	7
Obligate carriers^a^	-	12	-
Healthy Tyrolean Grey cattle	885	28	-
Healthy control cattle from 12 other breeds	28	-	-
Total	913	40	7

a Parents of affected offspring were classified as obligate carriers.

**Figure 5:**
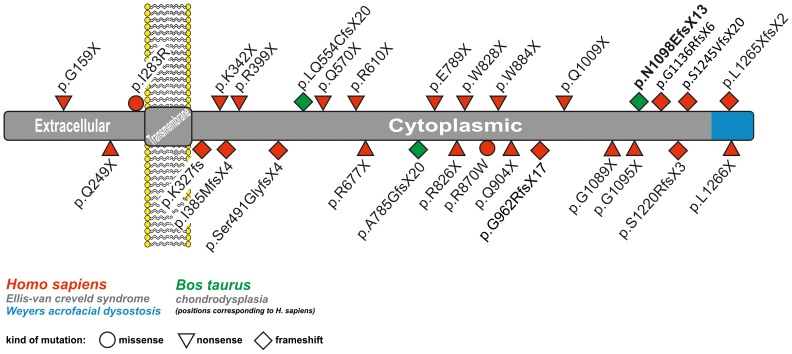
Localization of known human and bovine mutations affecting the EVC2 protein. The mutation found in chondrodysplastic Tyrolean Grey calves is shown in bold face. Previously reported mutations causing chondrodysplasia in cattle (shown in green) and Ellis-van Crefeld syndrome or Weyers acrofacial dysostosis (shown in red) in man are displayed with regard to their position and the cellular compartment of the protein [22], [25], [28], [36]–[38].

The mutation is predicted to cause a frameshift and premature stop codon beginning with amino acid residue 998 in the bovine EVC2 protein sequence (p.Asp998GlufsTer13). Although it is unclear whether the mutant protein is actually expressed, it is very unlikely that the mutant protein is able to fulfil its physiological function. Due to the frameshift and the premature stop codon, any mutant protein produced would be shortened and contain 13 amino acids different from the normal protein. These protein changes could potentially interfere with normal cellular function. Interestingly, chondrodysplastic dwarfism in Japanese Brown cattle, a highly similar phenotype, is also caused by *EVC2* mutations: one, a single nucleotide substitution leading to activation of a cryptic splicing donor site, and the other, a 1 bp deletion resulting in a frameshift mutation [11]. The mutations described in Japanese brown cattle lie in an earlier region compared to the one we report here (**Figure 5**) but has a comparable effect on limb development and a similar mechanism of transmission. In *Homo sapiens*, mutations in *EVC* and *EVC2* genes cause recessively inherited Ellis-van Creveld syndrome and dominantly inherited Weyers acrofacial dysostosis. [14], [18]–[22]. Weyers acrofacial dysostosis is associated with mutations in the last exon of EVC2, corresponding to the extreme cytoplasmic end of the protein and leading to impaired Hedgehog signaling (**Figure 5**) [23].

While expression of a truncated EVC2 protein (as occurs in Ellis-van Creveld syndrome) has been reported to not alter Hedgehog signaling in a NIH 3T3 cell line, it is thought that the mutated RNA could be subjected to decay or the truncated peptide could be degraded thus preventing its function [24]. The clinical features of human Ellis-van Creveld syndrome include skeletal malformations such as shortening of the limbs, short ribs and postaxial polydactyly; dysplastic nails are common and multiple frenulae are present in the mouth [25]. In about 60% of patients with Ellis-van Creveld syndrome cardiovascular malformations have been reported [26]. Mutations in *EVC2* are also usually associated with defects in the development of organs derived from ectoderm, such as teeth [24]. In human patients the clinical signs are more severe than those described in Japanese brown cattle, and several of the defects reported in humans were not seen in the Tyrolean Grey calves in this study. Patients with Ellis-van Creveld syndrome have been reported to have abnormal genitalia [27], in particular epispadias [28]. Similarly, affected Tyrolean Grey cattle also had abnormal genitalia, albeit in only in female calves, and the defect could be better defined as precocious growth. Given the known functions of the *EVC2* gene, and the effects of *EVC2* mutations in humans and cattle we conclude that the identified 2 bp deletion is causative for chondrodysplastic dwarfism in Tyrolean Grey cattle.

### Conclusions

In conclusion, the identification of a causative mutation for dwarfism in Tyrolean Grey cattle provides an additional naturally occurring animal model for human Ellis-van Creveld syndrome due to *EVC2* mutations. This study highlights the potential for combining the unique family structures of a livestock population, with the availability of SNP array genotyping and whole genome re-sequencing to determine the causative variant of a recessive disorder in just a couple of weeks. Approximately five to ten cases are sufficient to allow identification of associated markers and finally the causative mutation in an emerging outbreak of a genetic disease within a breed, on, thus allowing identification of carriers and selection against the deleterious allele.

## Material and Methods

### Ethics statement

All animal work was conducted according to national and international guidelines for animal welfare. There is no permit number as this study was not based on an invasive animal experiment and used naturally occurring cases. The samples used were taken from different cattle farms in Italy and all cattle owners agreed that the samples could be used in the study. Data was obtained during diagnostic procedures that would have been carried out anyway. This is a very special situation in veterinary medicine. As the data is from client-owned cattle that underwent veterinary exams, there was no “animal experiment” according to the legal definitions in Italy.

### Animals and genotyping

We collected blood samples from 7 affected calves from different farms. Genotyping of these cases was performed using the BovineHD BeadChip (Illumina), including 777,962 evenly distributed SNPs and standard protocols as recommended by the manufacturer. Additionally, we collected blood and semen samples from 12 cattle recorded as parents of affected offspring. Stored DNA from 913 healthy Tyrolean Grey cattle was also used, resulting in a total of 932 samples from this breed. For the mutation analysis we used 28 normal cattle from 12 genetically diverse *Bos taurus* breeds (**Table S2**).

### Histology

Samples of bone tissue from the femur, humerus and vertebrae were formalin-fixed, decalcified for 36 hours in Decalcifier for biopsy (Kaltek S.R.L.) followed by embedding in paraffin-wax. Specimens of uterus and ovaries were formalin-fixed and paraffin-wax embedded. Sections of organs and bones were then cut at 3–4 µm and stained with haematoxylin and eosin.

### Genome-wide association and homozygosity mapping

For the GWAS R Studio with the GenAbel package was used [29]. As a preliminary step in the analysis we performed a first quality control to remove markers and individuals with call rates <90% from the analysis. We also removed markers with minor allele frequency (MAF) <5% and markers strongly deviating from Hardy-Weinberg equilibrium (p<10^−6^). This was followed by a mixed model association study using the appropriate line of command in the Genabel package. The threshold of P≤0.01 for genome-wide significance was Bonferroni-adjusted to account for multiple testing (0.01/31,  = 3.14×10^−7^). A second, less stringent, adjustment of P≤0.1 was also performed. The software PLINK [30] was used to search for extended intervals of homozygosity with shared alleles as described previously. Individuals and SNPs were selected using the commands —keep, and —extract, while final files were generated through the —merge command. Homozygosity analysis was performed on all cases using the commands —cow, —homozyg and —homozyg-group.

### Whole genome re-sequencing

We prepared a fragment library with a 200 bp insert size and collected one lane of Illumina HiSeq2500 paired-end reads (2×100 bp); the fastq files were created using Casava 1.8. We obtained a total of 356,094,795 paired-end reads which were then mapped to the cow reference genome UMD3.1/bosTau6 and aligned using Burrows-Wheeler Aligner (BWA) version 0.5.9-r16 [31] with default settings. The mapping showed 313195442 reads had unique mapping positions giving roughly13x coverage. The SAM file generated by BWA was then converted to BAM and the reads sorted by chromosome using samtools [32]. PCR duplicates were marked using Picard tools (http://sourceforge.net/projects/picard/). We used the Genome Analysis Tool Kit (GATK version 2.4.9, [33]) to perform local realignment and to produce a cleaned BAM file. Variant calls were then made with the unified genotyper module of GATK. The variant data for each sample was obtained in variant call format (version 4.0) as raw calls for all samples and sites flagged using the variant filtration module of GATK. Variant filtration was performed, following best practice documentation of GATK version 4. The snpEFF software [34] together with the UMD3.1/bosTau Ensembl annotation was used to predict the functional effects of detected variants. The genome data has been made freely available under accession no. PRJEB5435 at the European Nucleotide Archive [35].

### Sanger sequencing

The associated variant was genotyped by re-sequencing of targeted PCR products using Sanger sequencing technology. PCR products were amplified using flanking primers (forward 5′-GGCCCAAGATGACATCAGTT-3′, reverse 5′-CTGTTGTTTTTGCTGCTGCT-3′) with AmpliTaqGold360Mastermix (Life Technologies) and the products directly sequenced using the PCR primers on an ABI 3730 capillary sequencer (Life Technologies) after treatment with exonuclease I and shrimp alkaline phosphatase. Sequence data were analyzed using Sequencher 5.1 (GeneCodes).

## Supporting Information

Figure S1 **GWAS (A): QQ-plot.** QQ- plots showing the observed versus expected log p-values. The diagonal line in the QQ plots indicates the distribution of SNP markers under the null hypothesis, and the skewing of a marker toward the upper side suggests that it has a stronger association with the “affected” condition than would be expected by mere chance. Note the deviation of observed values from the expected, indicating a consistent difference between cases and controls, reflecting the GWAS result obtained. (**B**) Detailed view of the significantly associated region of BTA 6. The zone evidenced by homozygosity mapping is marked in red.
(TIF)

Figure S2 **Changes of the female gential tract of a single chondrodysplastic animal.** (**A**) The uterus shows multiple endometrial polyps (indicated by arrows) which protrude into the lumen and the ovaries show cystic structures. (**B**) Histology of the uterus: multiple endometrial polyps, composed by a core of proliferating and edematous tissue lined by endometrial epithelium (H&E 4x).
(TIF)

Table S1 Top 20 most significantly associated SNPs.
(DOCX)

Table S2 List of all coding variants in the candidate region, before the filtering out of variants that do not cause alterations in the amino acid sequence, and exclusion of variants after comparison with other genomes.
(DOCX)

Table S3 Control cattle genomes.
(DOCX)
