# Correction: Deletion in the *EVC2* Gene Causes Chondrodysplastic Dwarfism in Tyrolean Grey Cattle

**DOI:** 10.1371/journal.pone.0102928

**Published:** 2014-07-11

**Authors:** The PLOS ONE Staff

Figure 1 is incorrect. Please see the correct Figure 1 here.

**Figure 1:**
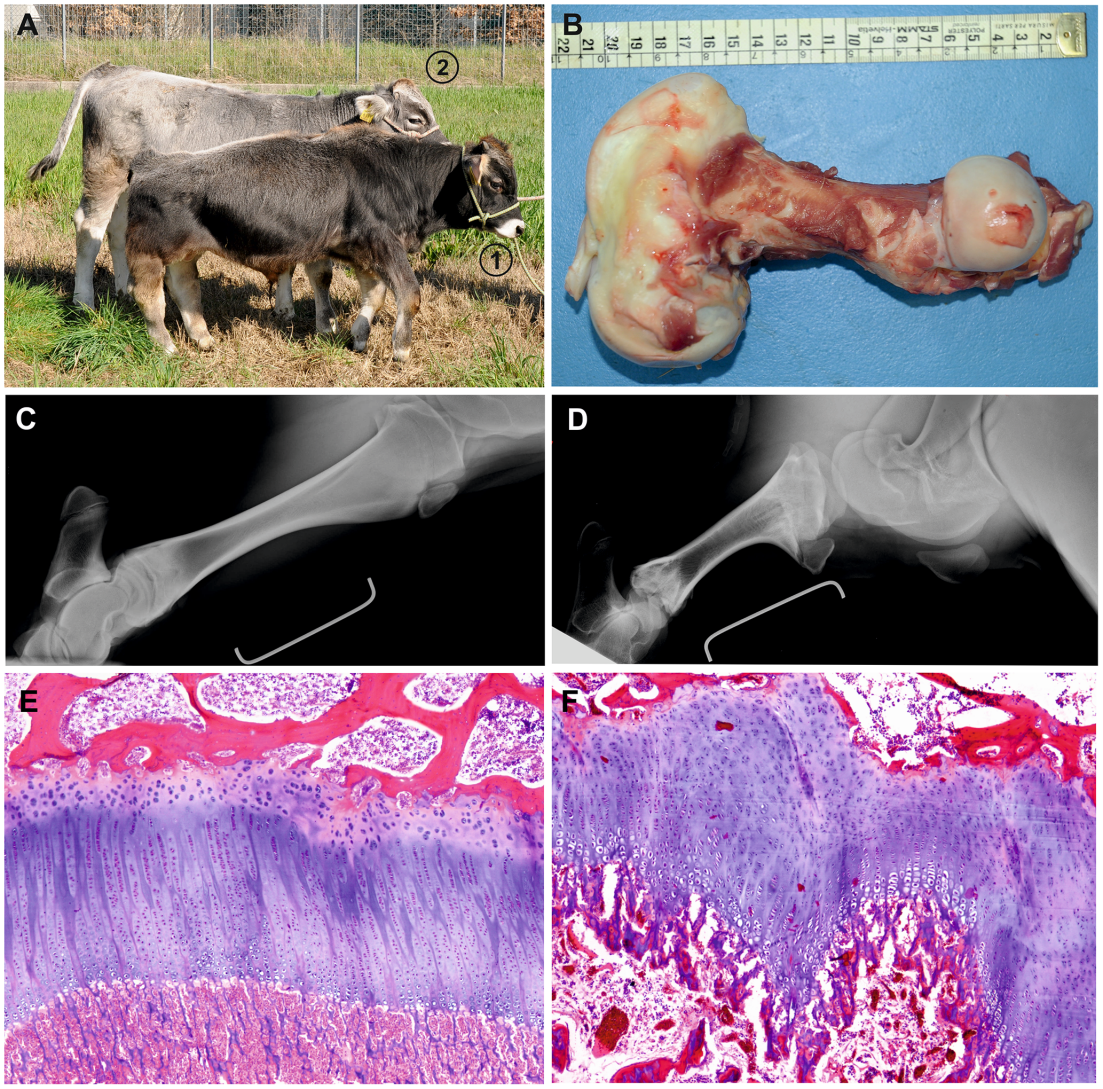
Dwarf Tyrolean Grey calf phenotype. (**A**) Seven month old affected Tyrolean Grey calf (1) and five month old normal control (2). Note the shorter legs and twisted posture. The head proportions and axial skeleton appear to be normal. At the moment the photo shoot both animals show an identical femur width of 3 cm and the distance between caput ossis femoris and trochlea ossis femoris was 20.9 cm for the affected and 26.8 cm in the control. (**B**) The femur is shortened and moderately twisted, with enlargement of, particularly the distal metaphysis and epiphysis. The scale is shown for reference. (**C+D**) X-ray of tibia from a normal and an affected animal. The ruler corresponds to 10 cm. (**E+F**) Histology of the femur of normally developed control of same age and an affected animal showing irregularity of the growth plate and increased thickness of the reserve zone (H&E 4x). Both the proliferative and hypertrophic zones are shortened and disorganized. Trabeculae in the primary spongiosa are also truncated.
