# Supplementary figures and images for: Deletion in the EVC2 Gene Causes Chondrodysplastic Dwarfism in Tyrolean Grey Cattle
Source: PLoS One. 2014 Apr 14;9(4):e94861. doi: 10.1371/journal.pone.0094861 (PMC3986253; doi:10.1371/journal.pone.0094861)

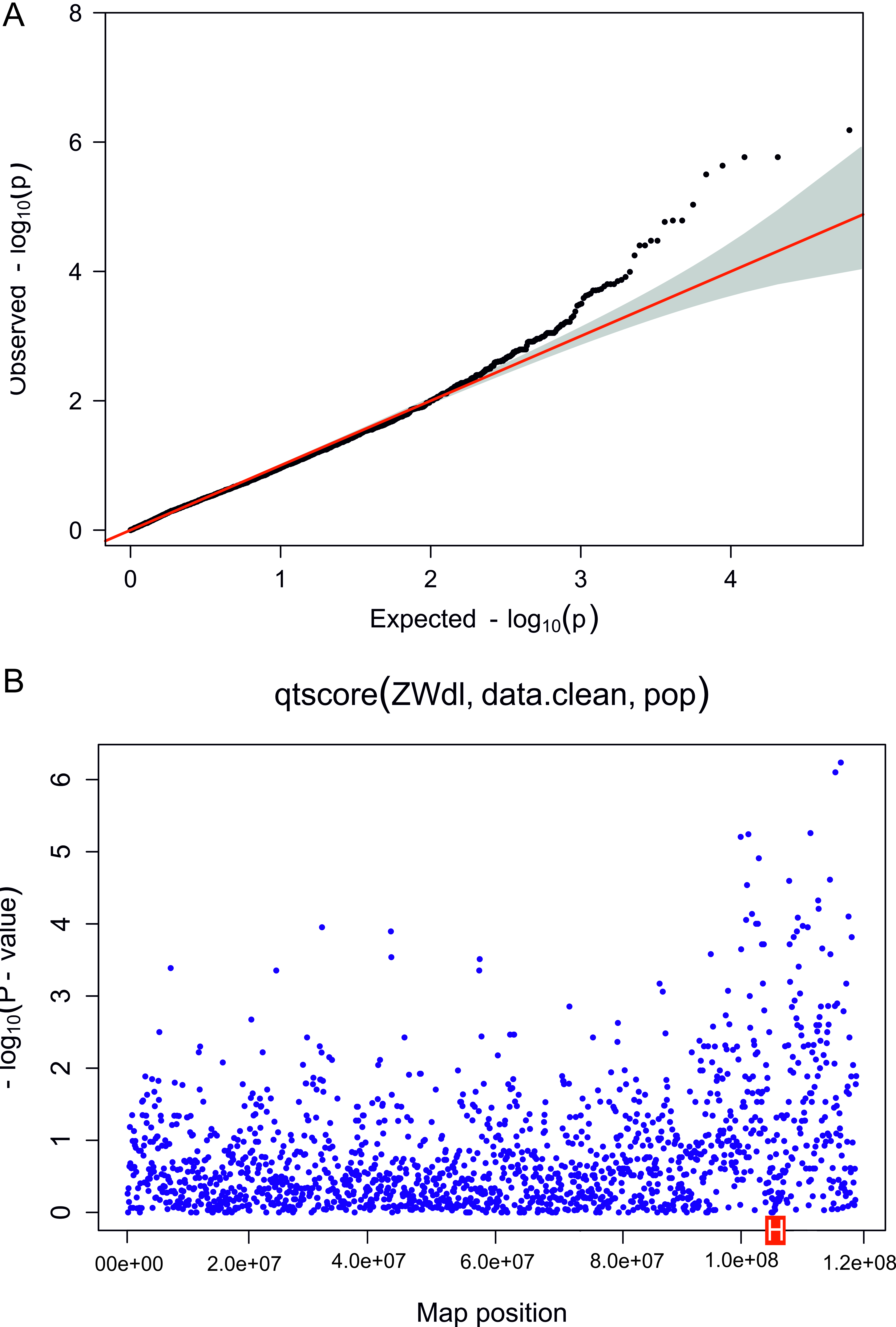

Supplement: Figure S1 — GWAS (A): QQ-plot. QQ- plots showing the observed versus expected log p-values. The diagonal line in the QQ plots indicates the distribution of SNP markers under the null hypothesis, and the skewing of a marker toward the upper side suggests that it has a stronger association with the “affected” condition than would be expected by mere chance. Note the deviation of observed values from the expected, indicating a consistent difference between cases and controls, reflecting the GWAS result obtained. (B) Detailed view of the significantly associated region of BTA 6. The zone evidenced by homozygosity mapping is marked in red. (TIF) [file pone.0094861.s001.tif]

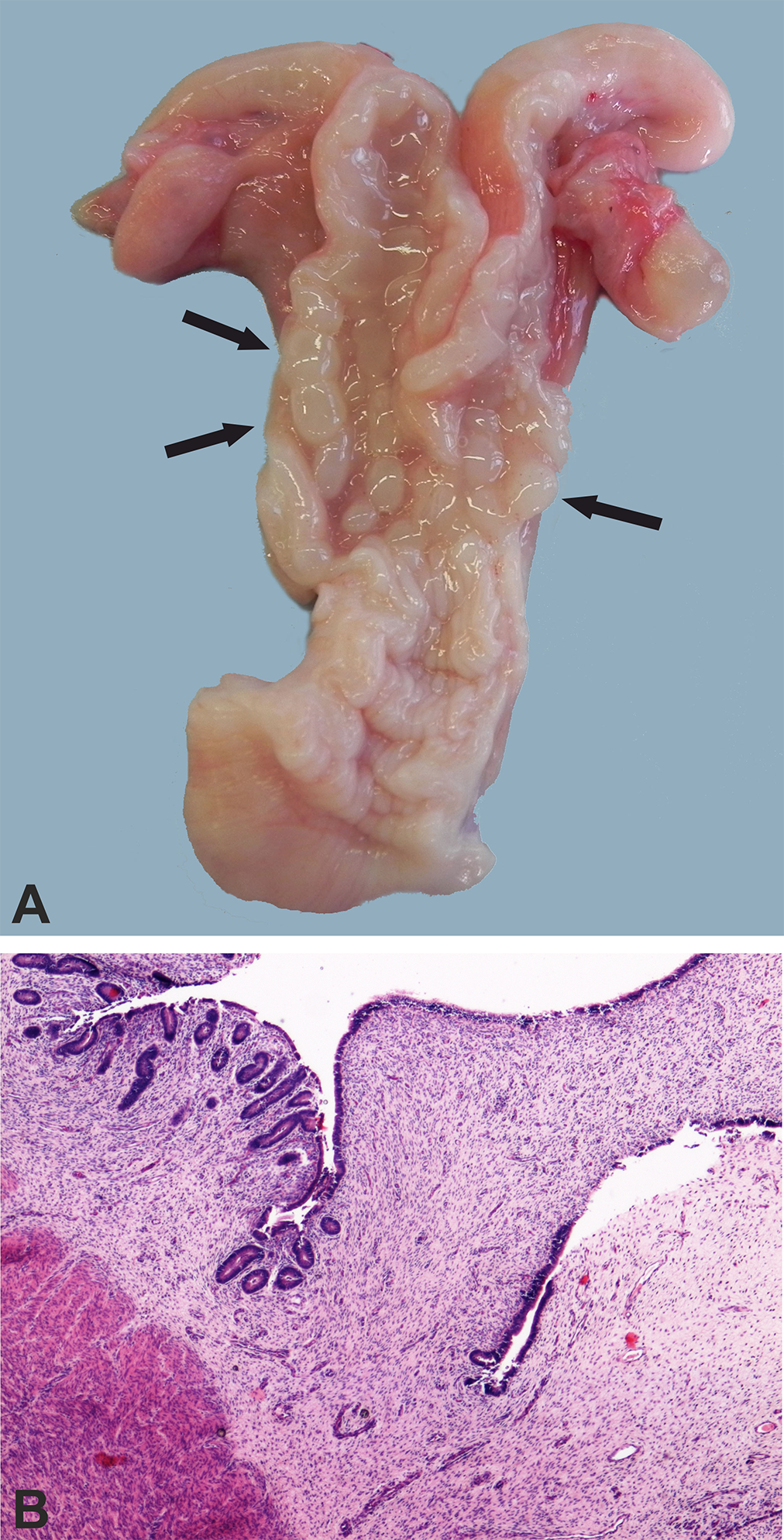

Supplement: Figure S2 — Changes of the female gential tract of a single chondrodysplastic animal. (A) The uterus shows multiple endometrial polyps (indicated by arrows) which protrude into the lumen and the ovaries show cystic structures. (B) Histology of the uterus: multiple endometrial polyps, composed by a core of proliferating and edematous tissue lined by endometrial epithelium (H&E 4x). (TIF) [file pone.0094861.s002.tif]
