# Supplementary material for: Deletion in the EVC2 Gene Causes Chondrodysplastic Dwarfism in Tyrolean Grey Cattle
Source: PLoS One. 2014 Apr 14;9(4):e94861. doi: 10.1371/journal.pone.0094861 (PMC3986253; doi:10.1371/journal.pone.0094861)
Supplement: Table S1 — Top 20 most significantly associated SNPs. (DOCX) [file pone.0094861.s003.docx]

**Table S1.** Top 20 most significantly associated SNPs.

| **SNP name^*^** | **Chromosome** | **Position^**^** | **effB^#^** | **se_effB^##^** | **chi2.1df^###^** | **P1df^####^** | **Pc1df^#####^** |
| --- | --- | --- | --- | --- | --- | --- | --- |
| ***HAPMAP42933BTA40454*** | 1 | 85731453 | 1 | 0.144279 | 48.03885 | 4.18E-12 | 4.03E-11 |
| ***ARSBFGLNGS27311*** | 6 | 116821583 | 0.742528 | 0.11886 | 39.02582 | 4.18E-10 | 2.66E-09 |
| ***BTB00652797*** | 16 | 65695370 | 0.556009 | 0.089717 | 38.40748 | 5.74E-10 | 3.54E-09 |
| ***BTB01619785*** | 6 | 115672242 | 0.383995 | 0.06739 | 32.46809 | 1.21E-08 | 5.68E-08 |
| ***HAPMAP54495RS29018810*** | 11 | 49612828 | 0.610067 | 0.108127 | 31.8335 | 1.68E-08 | 7.65E-08 |
| ***ARSBFGLNGS98530*** | 0 | unknown | 0.401566 | 0.071658 | 31.40421 | 2.10E-08 | 9.35E-08 |
| ***ARSBFGLNGS42004*** | 19 | 39268518 | 0.547099 | 0.111484 | 24.08255 | 9.23E-07 | 2.94E-06 |
| ***ARSBFGLNGS23835*** | 6 | 104385502 | 0.590094 | 0.120481 | 23.98877 | 9.69E-07 | 3.07E-06 |
| **BTB00671090** | 17 | 10525497 | 0.461019 | 0.094501 | 23.79921 | 1.07E-06 | 3.36E-06 |
| **BTB00886055** | 24 | 32449475 | 0.504066 | 0.104921 | 23.08052 | 1.55E-06 | 4.72E-06 |
| **HAPMAP43683BTA77737** | 6 | 105690770 | 0.276628 | 0.058175 | 22.61111 | 1.98E-06 | 5.89E-06 |
| **BTB01150784** | 17 | 41686505 | 0.521694 | 0.113073 | 21.28705 | 3.95E-06 | 1.11E-05 |
| **HAPMAP49224BTA57251** | 24 | 9058158 | 0.551272 | 0.122023 | 20.41015 | 6.25E-06 | 1.68E-05 |
| **BTB01845506** | 21 | 10125791 | 0.656806 | 0.147444 | 19.84352 | 8.40E-06 | 2.20E-05 |
| **ARSBFGLNGS3276** | 20 | 63394223 | 0.630073 | 0.142551 | 19.53637 | 9.87E-06 | 2.54E-05 |
| **ARSBFGLNGS106073** | 15 | 29821955 | 0.538336 | 0.122649 | 19.26533 | 1.14E-05 | 2.89E-05 |
| **HAPMAP26570BTC046391** | 6 | 103332668 | 0.254137 | 0.058124 | 19.11704 | 1.23E-05 | 3.11E-05 |
| **BTB00445816** | 10 | 94030802 | 0.690503 | 0.157972 | 19.10615 | 1.24E-05 | 3.12E-05 |
| **ARSBFGLNGS31749** | 24 | 4669101 | 0.444479 | 0.101834 | 19.05079 | 1.27E-05 | 3.21E-05 |
| **ARSBFGLNGS101995** | 19 | 53606174 | 0.376777 | 0.086447 | 18.99622 | 1.31E-05 | 3.29E-05 |

^*^Italic indicates being above the less stringent Bonferroni adjustment threshold. Underscored markers are above the stringent threshold of significance for Bonferroni adjustment.

^**^precise location of the SNP on the respective cattle chromosome.

^#^effB: Effect of the B allele in allelic test.

^##^se_effB standard error of the estimate of EffB.

^###^chi2.1df: Chi-square distribution of 1-d.f.

^####^P1df: corresponding list of P-values of 1-d.f. (additive or allelic) test for association between SNP and trait.

^#####^Pc1df: P-values from the 1-d.f. test for association between SNP and trait (statistics is corrected for possible inflation); 1-d.f. = 1 degree of freedom.
