# Supplementary material for: Deletion in the EVC2 Gene Causes Chondrodysplastic Dwarfism in Tyrolean Grey Cattle
Source: PLoS One. 2014 Apr 14;9(4):e94861. doi: 10.1371/journal.pone.0094861 (PMC3986253; doi:10.1371/journal.pone.0094861)
Supplement: Table S2 — List of all coding variants in the candidate region, before the filtering out of variants that do not cause alterations in the amino acid sequence, and exclusion of variants after comparison with other genomes. (DOCX) [file pone.0094861.s004.docx]

**Table S2.** List of all coding variants in the candidate region.

| **Position** | **Reference** | **Variant** | **Effect** | **Functional class** | **Codon change** | **Amino acid change** | **Gene** |
| --- | --- | --- | --- | --- | --- | --- | --- |
| 104917994 | C | T | INTRON |  |  |  | JKIP1_BOVIN |
| 104917994 | C | T | SYNONYMOUS_CODING | SILENT | aaC/aaT | N613 | JKIP1_BOVIN |
| 104918116 | TC | T | INTRON |  |  |  | JKIP1_BOVIN |
| 104918116 | TC | T | UTR_3_PRIME |  |  |  | JKIP1_BOVIN |
| 104921678 | G | C | DOWNSTREAM |  |  |  | JKIP1_BOVIN |
| 104921678 | G | C | NON_SYNONYMOUS_CODING | MISSENSE | gGg/gCg | G657A | JKIP1_BOVIN |
| 104991456 | TCAC | T | CODON_DELETION |  | cac/- | H23- | C4orf50 |
| 104991503 | C | T | NON_SYNONYMOUS_CODING | MISSENSE | gCg/gTg | A38V | C4orf50 |
| 104991520 | T | G | NON_SYNONYMOUS_CODING | MISSENSE | Tcc/Gcc | S44A | C4orf50 |
| 104991528 | C | T | SYNONYMOUS_CODING | SILENT | ctC/ctT | L46 | C4orf50 |
| 104991549 | G | A | SYNONYMOUS_CODING | SILENT | aaG/aaA | K53 | C4orf50 |
| 104994966 | G | A | SYNONYMOUS_CODING | SILENT | aaG/aaA | K59 | C4orf50 |
| 104998324 | T | A | INTRON |  |  |  | C4orf50 |
| 104998746 | C | T | SYNONYMOUS_CODING | SILENT | ctC/ctT | L106 | C4orf50 |
| 104998761 | G | A | SYNONYMOUS_CODING | SILENT | acG/acA | T111 | C4orf50 |
| 104998765 | A | G | NON_SYNONYMOUS_CODING | MISSENSE | Atc/Gtc | I113V | C4orf50 |
| 105010542 | C | G | INTRON |  |  |  | C4orf50 |
| 105014969 | C | T | NON_SYNONYMOUS_CODING | MISSENSE | cCc/cTc | P196L | C4orf50 |
| 105015064 | G | A | NON_SYNONYMOUS_CODING | MISSENSE | Gga/Aga | G228R | C4orf50 |
| 105015184 | G | A | NON_SYNONYMOUS_CODING | MISSENSE | Gtc/Atc | V268I | C4orf50 |
| 105105393 | A | G | UTR_5_PRIME |  |  |  | CRMP1 |
| 105105520 | G | T | UTR_5_PRIME |  |  |  | CRMP1 |
| 105130813 | C | T | NON_SYNONYMOUS_CODING | MISSENSE | aCg/aTg | T84M | CRMP1 |
| 105130859 | C | T | SYNONYMOUS_CODING | SILENT | ggC/ggT | G99 | CRMP1 |
| 105132099 | T | C | INTRON |  |  |  | CRMP1 |
| 105146567 | A | G | SYNONYMOUS_CODING | SILENT | acA/acG | T218 | CRMP1 |
| 105148444 | C | T | SYNONYMOUS_CODING | SILENT | agC/agT | S259 | CRMP1 |
| 105152565 | C | T | SYNONYMOUS_CODING | SILENT | gaC/gaT | D286 | CRMP1 |
| 105152634 | G | A | SYNONYMOUS_CODING | SILENT | ccG/ccA | P309 | CRMP1 |
| 105156701 | G | A | SYNONYMOUS_CODING | SILENT | gcG/gcA | A339 | CRMP1 |
| 105166647 | C | T | UTR_3_PRIME |  |  |  | CRMP1 |
| 105166693 | T | C | UTR_3_PRIME |  |  |  | CRMP1 |
| 105166705 | C | T | UTR_3_PRIME |  |  |  | CRMP1 |
| 105171711 | C | T | DOWNSTREAM |  |  |  | CRMP1 |
| 105171711 | C | T | DOWNSTREAM |  |  |  | EVC |
| 105171711 | C | T | INTERGENIC |  |  |  |  |
| 105171907 | C | T | UTR_3_PRIME |  |  |  | EVC |
| 105173878 | T | G | NON_SYNONYMOUS_CODING | MISSENSE | gAa/gCa | E916A | EVC |
| 105175035 | C | T | NON_SYNONYMOUS_CODING | MISSENSE | Gcg/Acg | A876T | EVC |
| 105175090 | C | A | SYNONYMOUS_CODING | SILENT | gcG/gcT | A857 | EVC |
| 105181122 | C | A | NON_SYNONYMOUS_CODING | MISSENSE | Gcc/Tcc | A847S | EVC |
| 105181147 | G | A | SYNONYMOUS_CODING | SILENT | gcC/gcT | A838 | EVC |
| 105184384 | G | C | NON_SYNONYMOUS_CODING | MISSENSE | aCg/aGg | T770R | EVC |
| 105208530 | G | A | SYNONYMOUS_CODING | SILENT | taC/taT | Y524 | EVC |
| 105225910 | C | T | NON_SYNONYMOUS_CODING | MISSENSE | Gtg/Atg | V516M | EVC |
| 105231425 | T | C | SYNONYMOUS_CODING | SILENT | gaA/gaG | E419 | EVC |
| 105231454 | C | T | NON_SYNONYMOUS_CODING | MISSENSE | Gag/Aag | E410K | EVC |
| 105231567 | G | A | NON_SYNONYMOUS_CODING | MISSENSE | aCg/aTg | T372M | EVC |
| 105242905 | C | T | SYNONYMOUS_CODING | SILENT | aaG/aaA | K264 | EVC |
| 105257188 | T | C | SYNONYMOUS_CODING | SILENT | gcA/gcG | A226 | EVC |
| 105258704 | A | G | SYNONYMOUS_CODING | SILENT | tgT/tgC | C204 | EVC |
| 105258776 | T | C | SYNONYMOUS_CODING | SILENT | tcA/tcG | S180 | EVC |
| 105258896 | G | A | SYNONYMOUS_CODING | SILENT | caC/caT | H140 | EVC |
| 105258939 | A | G | INTRON |  |  |  | EVC |
| 105261105 | G | A | SYNONYMOUS_CODING | SILENT | taC/taT | Y102 | EVC |
| 105261112 | TG | T | INTRON |  |  |  | EVC |
| 105268222 | G | A | SYNONYMOUS_CODING | SILENT | gaC/gaT | D100 | EVC |
| 105268240 | C | T | SYNONYMOUS_CODING | SILENT | aaG/aaA | K94 | EVC |
| 105268353 | C | A | INTRON |  |  |  | EVC |
| 105291633 | C | T | SYNONYMOUS_CODING | SILENT | tcC/tcT | S26 | EVC2 |
| 105291715 | A | G | INTRON |  |  |  | EVC2 |
| 105297790 | C | T | SYNONYMOUS_CODING | SILENT | tcC/tcT | S101 | EVC2 |
| 105297875 | A | G | NON_SYNONYMOUS_CODING | MISSENSE | Agg/Ggg | R130G | EVC2 |
| 105303440 | C | G | SYNONYMOUS_CODING | SILENT | tcC/tcG | S141 | EVC2 |
| 105303512 | G | C | SYNONYMOUS_CODING | SILENT | ctG/ctC | L165 | EVC2 |
| 105333721 | G | A | NON_SYNONYMOUS_CODING | MISSENSE | tGc/tAc | C185Y | EVC2 |
| 105333803 | C | T | SYNONYMOUS_CODING | SILENT | caC/caT | H198 | EVC2 |
| 105336656 | G | A | NON_SYNONYMOUS_CODING | MISSENSE | Gct/Act | A259T | EVC2 |
| 105336691 | C | T | SYNONYMOUS_CODING | SILENT | tcC/tcT | S270 | EVC2 |
| 105336699 | C | T | NON_SYNONYMOUS_CODING | MISSENSE | cCc/cTc | P273L | EVC2 |
| 105373933 | C | T | SYNONYMOUS_CODING | SILENT | ttC/ttT | F450 | EVC2 |
| 105377761 | A | T | INTRON |  |  |  | EVC2 |
| 105377761 | A | T | SPLICE_SITE_ACCEPTOR |  |  |  | EVC2 |
| 105377905 | C | T | NON_SYNONYMOUS_CODING | MISSENSE | gCg/gTg | A518V | EVC2 |
| 105389780 | G | A | DOWNSTREAM |  |  |  | U6 |
| 105389780 | G | A | SYNONYMOUS_CODING | SILENT | ctG/ctA | L712 | EVC2 |
| 105394421 | C | T | SYNONYMOUS_CODING | SILENT | tcC/tcT | S746 | EVC2 |
| 105394466 | C | T | SYNONYMOUS_CODING | SILENT | ttC/ttT | F761 | EVC2 |
| 105420748 | A | G | SYNONYMOUS_CODING | SILENT | caA/caG | Q856 | EVC2 |
| 105420778 | C | T | SYNONYMOUS_CODING | SILENT | gcC/gcT | A866 | EVC2 |
| 105420811 | A | G | SYNONYMOUS_CODING | SILENT | gcA/gcG | A877 | EVC2 |
| 105429805 | A | G | INTRON |  |  |  | EVC2 |
| 105431679 | C | A | INTRON |  |  |  | EVC2 |
| 105431702 | GAC | G | FRAME_SHIFT |  | - | -1000 | EVC2 |
| 105437043 | C | T | INTRON |  |  |  | EVC2 |
| 105437148 | C | T | SYNONYMOUS_CODING | SILENT | gcC/gcT | A1055 | EVC2 |
| 105437226 | G | A | SYNONYMOUS_CODING | SILENT | caG/caA | Q1081 | EVC2 |
| 105451459 | A | G | NON_SYNONYMOUS_CODING | MISSENSE | Aga/Gga | R1141G | EVC2 |
| 105451494 | T | C | SYNONYMOUS_CODING | SILENT | gaT/gaC | D1152 | EVC2 |
| 105451506 | A | G | SYNONYMOUS_CODING | SILENT | ccA/ccG | P1156 | EVC2 |
| 105451509 | G | A | SYNONYMOUS_CODING | SILENT | gtG/gtA | V1157 | EVC2 |
| 105451513 | A | G | NON_SYNONYMOUS_CODING | MISSENSE | Att/Gtt | I1159V | EVC2 |
| 105451590 | G | A | SYNONYMOUS_CODING | SILENT | gaG/gaA | E1184 | EVC2 |
| 105451613 | GGAA | G | CODON_DELETION |  | aggaag/agg | RK1192R | EVC2 |
| 105451666 | T | A | NON_SYNONYMOUS_CODING | MISSENSE | Ttg/Atg | L1210M | EVC2 |
| 105451707 | G | A | UTR_3_PRIME |  |  |  | EVC2 |
| 105451735 | G | A | UTR_3_PRIME |  |  |  | EVC2 |
| 105451855 | C | T | UTR_3_PRIME |  |  |  | EVC2 |
| 105451872 | T | C | UTR_3_PRIME |  |  |  | EVC2 |
| 105451875 | G | A | UTR_3_PRIME |  |  |  | EVC2 |
| 105451938 | C | T | UTR_3_PRIME |  |  |  | EVC2 |
| 105451939 | G | A | UTR_3_PRIME |  |  |  | EVC2 |
| 105451955 | C | G | UTR_3_PRIME |  |  |  | EVC2 |
| 105451968 | A | AC | UTR_3_PRIME |  |  |  | EVC2 |
| 105451982 | G | A | UTR_3_PRIME |  |  |  | EVC2 |
| 105452043 | G | A | UTR_3_PRIME |  |  |  | EVC2 |
| 105452047 | C | T | UTR_3_PRIME |  |  |  | EVC2 |
| 105452053 | T | C | DOWNSTREAM |  |  |  | EVC2 |
| 105452053 | T | C | INTERGENIC |  |  |  |  |
| 105518227 | C | T | SYNONYMOUS_CODING | SILENT | aaG/aaA | K142 | STK32B |
| 105524219 | G | T | SYNONYMOUS_CODING | SILENT | gtC/gtA | V56 | STK32B |
| 105524312 | G | A | SYNONYMOUS_CODING | SILENT | taC/taT | Y25 | STK32B |
| 105911157 | G | A | EXON |  |  |  | 5S_rRNA |
| 105926927 | C | T | NON_SYNONYMOUS_CODING | MISSENSE | Cgg/Tgg | R22W | CYTL1 |
| 105929706 | G | A | NON_SYNONYMOUS_CODING | MISSENSE | cGg/cAg | R76Q | CYTL1 |
| 106061690 | A | C | UTR_3_PRIME |  |  |  | F1N4A2_BOVIN |
| 106061690 | A | AC | UTR_3_PRIME |  |  |  | F1N4A2_BOVIN |
| 106061701 | A | C | UTR_3_PRIME |  |  |  | F1N4A2_BOVIN |
| 106061787 | C | G | UTR_3_PRIME |  |  |  | F1N4A2_BOVIN |
| 106061791 | T | G | UTR_3_PRIME |  |  |  | F1N4A2_BOVIN |
| 106062270 | C | T | SYNONYMOUS_CODING | SILENT | gcG/gcA | A286 | F1N4A2_BOVIN |
| 106062665 | G | A | INTRON |  |  |  | F1N4A2_BOVIN |
| 106065385 | C | A | NON_SYNONYMOUS_CODING | MISSENSE | gaG/gaT | E47D | F1N4A2_BOVIN |
| 106065407 | A | G | NON_SYNONYMOUS_CODING | MISSENSE | gTg/gCg | V40A | F1N4A2_BOVIN |
| 106065409 | A | C | SYNONYMOUS_CODING | SILENT | acT/acG | T39 | F1N4A2_BOVIN |
| 106065591 | T | A | START_GAINED |  |  |  | F1N4A2_BOVIN |
| 106113754 | G | C | EXON |  |  |  | 5S_rRNA |
| 106365432 | T | C | SYNONYMOUS_CODING | SILENT | cgT/cgC | R40 | STX18 |
| 106447215 | T | C | INTRON |  |  |  | STX18 |
| 106447218 | T | C | INTRON |  |  |  | STX18 |
| 106460328 | T | A | NON_SYNONYMOUS_CODING | MISSENSE | Tat/Aat | Y203N | STX18 |
| 106481334 | C | T | DOWNSTREAM |  |  |  | Q08DZ1_BOVIN |
| 106481334 | C | T | UTR_3_PRIME |  |  |  | STX18 |
| 106481901 | G | A | DOWNSTREAM |  |  |  | Q08DZ1_BOVIN |
| 106481901 | G | A | UTR_3_PRIME |  |  |  | STX18 |
| 106482471 | TAAAA | TAAAAA,T | DOWNSTREAM |  |  |  | Q08DZ1_BOVIN |
| 106482471 | TAAAA | TAAAAA,T | DOWNSTREAM |  |  |  | Q08DZ1_BOVIN |
| 106482471 | TAAAA | TAAAAA,T | UTR_3_PRIME |  |  |  | STX18 |
| 106482471 | TAAAA | TAAAAA,T | UTR_3_PRIME |  |  |  | STX18 |
| 106482623 | T | A | DOWNSTREAM |  |  |  | Q08DZ1_BOVIN |
| 106482623 | T | A | UTR_3_PRIME |  |  |  | STX18 |
| 106482645 | GA | G | DOWNSTREAM |  |  |  | Q08DZ1_BOVIN |
| 106482645 | GA | G | UTR_3_PRIME |  |  |  | STX18 |
| 106482647 | A | G | DOWNSTREAM |  |  |  | Q08DZ1_BOVIN |
| 106482647 | A | G | UTR_3_PRIME |  |  |  | STX18 |
| 106482707 | C | T | DOWNSTREAM |  |  |  | Q08DZ1_BOVIN |
| 106482707 | C | T | UTR_3_PRIME |  |  |  | STX18 |
| 106483775 | C | T | DOWNSTREAM |  |  |  | STX18 |
| 106483775 | C | T | NON_SYNONYMOUS_CODING | MISSENSE | Gtt/Att | V167I | Q08DZ1_BOVIN |
| 106483798 | C | T | DOWNSTREAM |  |  |  | STX18 |
| 106483798 | C | T | NON_SYNONYMOUS_CODING | MISSENSE | cGc/cAc | R159H | Q08DZ1_BOVIN |

The results shown are before the filtering out of variants that do not cause alterations in the amino acid sequence, and exclusion of variants with the comparison with other genomes.
