# Supplementary material for: Deletion in the EVC2 Gene Causes Chondrodysplastic Dwarfism in Tyrolean Grey Cattle
Source: PLoS One. 2014 Apr 14;9(4):e94861. doi: 10.1371/journal.pone.0094861 (PMC3986253; doi:10.1371/journal.pone.0094861)
Supplement: Table S3 — Control cattle genomes. (DOCX) [file pone.0094861.s005.docx]

**Table S3.** Control cattle genomes.

| Sample Name | Gender | Breed |
| --- | --- | --- |
| Control 1 | f | Simmental |
| Control 2 | f | Holstein |
| Control 3 | f | Simmental |
| Control 4 | f | Simmental |
| Control 5 | f | Scotish Highland Cattle |
| Control 6 | f | Brown Swiss |
| Control 7 | f | Brown Swiss |
| Control 8 | f | Pezzata Rossa Italiana |
| Control 9 | f | Holstein x Simmental |
| Control 10 | m | Holstein |
| Control 11 | f | Holstein |
| Control 12 | f | Charolais |
| Control 13 | f | Hereford |
| Control 14 | f | Scotish Highland Cattle |
| Control 15 | f | Eringer |
| Control 16 | f | Brown Swiss |
| Control 17 | f | Holstein |
| Control 18 | f | Holstein |
| Control 19 | f | Brown Swiss |
| Control 20 | m | Belted Galloway |
| Control 21 | f | Eringer |
| Control 22 | f | Romagnola |
| Control 23 | f | Holstein |
| Control 24 | f | Holstein |
| Control 25 | f | Limousin x Holstein |
| Control 26 | f | Holstein |
| Control 27 | m | Limousin |
| Control 28 | m | Belted Galloway |
